# Possible quantum critical behavior revealed by the critical current density of hole doped high-*T*_c_ cuprates in comparison to heavy fermion superconductors

**DOI:** 10.1038/s41598-019-51467-4

**Published:** 2019-10-16

**Authors:** S. H. Naqib, R. S. Islam

**Affiliations:** Department of Physics, University of Rajshahi, Rajshahi, 6205 Bangladesh

**Keywords:** Materials science, Superconducting properties and materials

## Abstract

The superconducting critical current density, *J*_c_, in hole doped cuprates show strong dependence on the doped hole content, *p*, within the copper oxide plane(s). The doping dependent *J*_c_ mainly exhibits the variation of the intrinsic depairing critical current density as *p* is varied. *J*_c_(*p*) tends to peak at *p* ~ 0.185 in copper oxide superconductors. This particular value of the hole content, often termed as the critical hole concentration, has several features putative to a quantum critical point (QCP). Very recently, the pressure dependences of the superconducting transition temperature (*T*_c_) and the critical current (*I*_c_) in pure CeRhIn_5_ and Sn doped CeRhIn_5_ heavy fermion compounds have been reported (Nature Communications (2018) 9:44, 10.1038/s41467-018-02899-5). The critical pressure demarcates an antiferromagnetic quantum critical point where both *T*_c_ and *I*_c_ are maximized. We have compared and contrasted this behavior with those found for Y_1−*x*_Ca_*x*_Ba_2_Cu_3_O_7−*δ*_ in this brief communication. The resemblance of the systematic behavior of the critical current with pressure and hole content between heavy fermion systems and hole doped cuprates is significant. This adds to the circumstantial evidence that quantum critical physics probably plays a notable role behind the unconventional normal and superconducting state properties of copper oxide superconductors.

## Introduction

It has been well over three decades since the discovery of superconductivity at high transition temperature in hole doped copper oxide materials in the mid-eighties^1,2^. The precise mechanism leading to Cooper pairing of the extra holes added to the CuO_2_ planes of these strongly correlated electronic systems remains elusive till date^3–5^. A remarkable set of coexisting and often competing electronic ground states^3–7^ in hole doped cuprates pose a serious theoretical challenge which the condensed matter physics community is yet to surmount. The standard theory for condensed matter physics, the Landau Fermi-liquid theory, breaks down completely in the case of underdoped (UD) cuprates^3–5^. The overdoped (OD) side is comparatively more conventional but still exhibits a number of anomalous characteristics^8^.

In the absence of any agreed upon theoretical scheme to describe the Mott physics of undoped antiferromagnetic (AFM) insulating state and its eventual transformation to the pseudogapped normal state, charge and spin density ordered states, and *d*-wave superconductivity upon hole doping, in a coherent fashion, the cuprate research community has focused their attention in exploring various possible scaling relations and generic features found in these materials and in other strongly correlated electronic systems with non-Fermi liquid features^9–17^. These systematic studies of generic behaviors can provide us with important clues to unlock the mystery of the physics of electronic phase diagram of high-*T*_c_ cuprates in the normal and superconducting (SC) states.

Since late 1990s, it had been proposed that presence of a quantum critical point (QCP) in the *T*-*p* phase diagram could be responsible for unconventional charge and magnetic excitations that could possibly offer explanations for non Fermi-liquid like charge and magnetic transport properties of high *T*_c_ cuprates. The parameter *p* (often termed simply as *hole content* or *hole concentration*) signifies the number of doped holes per Cu atom in the CuO_2_ plane. A QCP is understood via the concept of quantum phase transition (QPT). Unlike ordinary phase transitions driven by thermal energy, a QPT is characterized at a particular value of non-thermal parameter (e.g., critical hole content for cuprates) where a continuous phase transition takes place between a quantum disordered phase and a quantum ordered phase at zero temperature. The correlations at the QCP demonstrate spatio-temporal scale invariance. This implies that the poles present in the quasiparticle (QP) spectral function as predicted in the Fermi-liquid theory are absent here. Instead, one finds a power-law scaling behavior and the QP spectral function assumes a form given by (*ω*/*T*), where *ω* sets the energy scale of the quantum critical excitation mode. This leads to a dissipative QP relaxation time given by h/(2π*k*_B_*T*) which in turn implies that the scattering QP rate is linear in *T*. Moving away from the QCP, the energy scale for which scale invariance is valid, gradually increases^18^. It is worth mentioning that this *T*-linear QP scattering rate is considered as one of the prime signature of possible quantum criticality in hole doped cuprates^11–14^ and other systems^18^.

Quite interestingly the singular interactions arising from the competing phases at the QCP can provide with the ‘glue’ for Cooper pairing at high temperatures^19–21^. For example, Castellani *et al*.^19^ presented a scenario where a QCP due to formation of incommensurate charge density waves roughly accounts for some of the generic features of the high-*T*_c_ cuprates, both in the normal and in the SC states, including a *d*-wave SC order parameter.

It should be mentioned that the presence and precise nature of a QCP in hole doped cuprates are hotly debated issues^22^. The situation is clearer in the case of heavy fermion (HF) and iron pnictide superconductors^22–24^. As far as SC HFs are concerned, CeCu_2_Si_2_ is the prime candidate for antiparamagnon mediated superconductivity near a spin density wave QCP^24^. Besides, superconductivity may emerge from the proximity to a magnetic field-induced QCP, like that in CeCoIn_5_^25^ and perhaps also in UBe_13_^26^. The case for QCP in iron pnictides is quite strong. Evidence for superconductivity in at least one iron pnictide due to AFM quantum critical spin fluctuation is overwhelming^22,27^. Identification of the ground electronic state, its symmetry and thermodynamic signature of the symmetry breaking at the QCP in cuprates, on the other hand, are unclear^22^ at the moment. Under the circumstances, a useful strategy is to compare and contrast various non-Fermi liquid like properties of cuprates with those of the HF and iron pnictide systems.

Very recently, Jung *et al*.^28^ have studied the SC critical currents, *I*_c_, in CeRhIn_5_ and 4.4% Sn-doped CeRhIn_5_ HF superconductors as a function of pressure (*P*). The *I*_c0_s (zero-field critical currents) of these HF compounds under pressure exhibit a universal temperature dependence, underlining that the peak in zero-field *I*_c0_(*P*) is determined predominantly by quantum critical fluctuations associated with a hidden magnetic QCP at a critical pressure *P*_c_, where superconducting transition temperature is also maximum. Motivated by this particular study^28^, we have investigated the hole content dependent zero-field critical current density, *J*_c0_, of a series of Y_1−*x*_Ca_*x*_Ba_2_Cu_3_O_7−*δ*_ superconductors over wide range of compositions. We have also looked at the hole content dependent vortex activation energy and irreversibility field of YBa_2_CuO_7−δ_ thin films in this investigation. The generic behaviors of the superconducting critical current density and vortex pinning characteristics in Y_1−*x*_Ca_*x*_Ba_2_Cu_3_O_7−*δ*_ and Ce-based HF superconductors show strikingly similar behavior. We have discussed this feature and their possible implication in this short communication. This is the first comparative systematic study based on critical current density between hole doped cuprates and heavy fermion superconductors to the best of our knowledge.

The rest of the paper is organized as follows. A brief description of Y_1−*x*_Ca_*x*_Ba_2_Cu_3_O_7−*δ*_ compounds and some details regarding the previous *J*_c_ and magnetic field dependent resistivity measurements are presented in Section 2. The results are presented and compared to those obtained for Ce-based HF superconductors in Section 3. Section 4 comprises of the discussion on the results and important conclusions of this study.

## Y_1−*x*_Ca_*x*_Ba_2_Cu_3_O_7−*δ*_ samples and measurements

**Figure 1:**
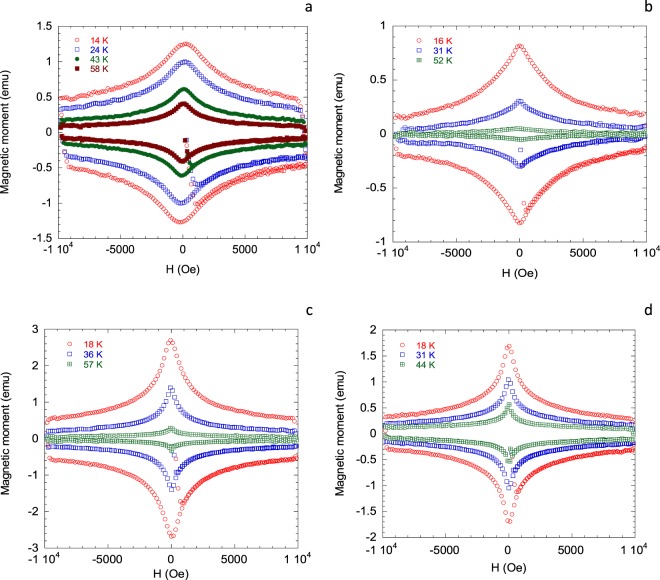
Representative magnetization loops for Y_1−*x*_Ca_*x*_Ba_2_Cu_3_O_7−*δ*_ thin films with different compositions at different temperatures. The magnetic field was applied along *c*-direction. The sample compositions and hole contents are (**a**) YBa_2_Cu_3_O_7−*δ*_; *p* = 0.162, (**b**) Y_0.95_Ca_0.05_Ba_2_Cu_3_O_7−*δ*_; p = 0.123, (**c**) Y_0.90_Ca_0.10_Ba_2_Cu_3_O_7−*δ*_; p = 0.198, and (**d**) Y_0.80_Ca_0.20_Ba_2_Cu_3_O_7−*δ*_; p = 0.144. *p*-values are accurate within ±0.004. For clarity only one in twenty data points are shown.

**Figure 2:**
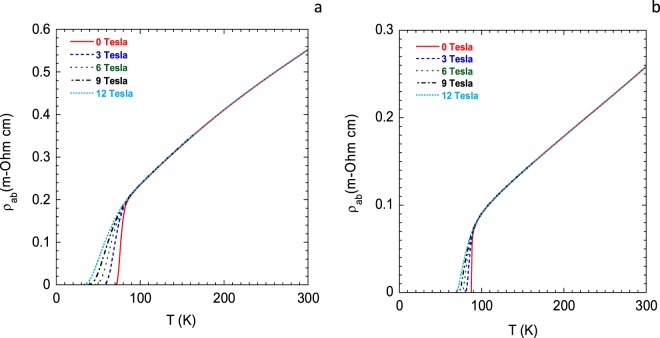
Representative magnetic field-dependent in-plane resistivity data for YBa_2_Cu_3_O_7−*δ*_ thin films with different hole contents. The magnetic fields were applied along the *c*-direction. The hole contents are (**a**) 0.118 and (**b**) 0.170. These values are accurate within ±0.004.

High-quality *c*-axis oriented crystalline thin films of Y_1−*x*_Ca_*x*_Ba_2_Cu_3_O_7−*δ*_ (*x* = 0.00, 0.05, 0.10, 0.20) were grown on SrTiO_3_ substrates using the method of pulsed LASER ablation technique. Substrates of dimensions 5 × 5 × 1 mm^3^ and 10 × 5 × 1 mm^3^ were used. The thicknesses of the films lie within 2800 ± 300 Å. Details regarding the film preparation and characterization can be found in ref.^29^. Hole content within the CuO_2_ planes were varied by two independent means. The oxygen deficiency, *δ*, in the CuO_1−*δ*_ chains were controlled via oxygen annealing at different temperatures and partial pressures. The Ca content, *x*, substituted for the Y atom in the charge reservoir layer also adds holes to the CuO_2_ planes independent of the oxygen loading in the CuO_1−*δ*_ chains. This enables one to access the overdoped side relatively easily. Pure YBCO with fully oxygen loaded CuO chains can give a maximum *p* value ~ 0.180. Information about the annealing treatments and magnetization measurements of the thin films can be found in refs^29–31^. The hole content was estimated with high degree of accuracy from three different methods: room temperature thermopower (*S*[290 K])^32,33^, *c*-axis lattice constant^30^, and the well known parabolic *T*_c_(*p*) relation^34,35^. In this paper we have used the *p*-values obtained from the *S*[290 K] data. This is quite insensitive to the crystalline order and disorder content of the sample and depends solely on the number of doped holes in the CuO_2_ plane. Details regarding the magnetic field dependent resistivity (*ρ*_*ab*_(*H*, *T*)) measurements and analysis of the flux dynamics can be found in ref.^36^. All the measurements presented for Y_1−*x*_Ca_*x*_Ba_2_Cu_3_O_7−*δ*_ in this study were done for the *H* II *c* configuration, where the supercurrent circulated in the CuO_2_ plane. We have shown representative M-H loops for Y_1−*x*_Ca_*x*_Ba_2_Cu_3_O_7−*δ*_ thin films at different temperatures and hole contents in Fig. 1. Representative *ρ*_*ab*_(*H*, *T*) data for YBa_2_Cu_3_O_7−*δ*_ thin films are shown in Fig. 2.

The zero-field critical current, *J*_c0_, for the Y_1−*x*_Ca_*x*_Ba_2_Cu_3_O_7−*δ*_ thin films with different amounts of Ca and oxygen deficiencies were calculated from the width of the magnetization loops at *H* = 0 G and the dimensions of the thin films following the method developed by Brandt and Indenbom^37^ for finite geometry with the modified critical state model.

## Hole Content Dependent Critical Current Density

The critical current density depends strongly on temperature. In this study, we have used the zero temperature critical current density for comparison. This was done by fitting the hole content dependent zero-field critical current density to the following relation^36,38^

1 $${J}_{c0}(t)={J}_{0}{(1-t)}^{n}$$

where, *t* = (*T*/*T*_*c*_), is the reduced temperature and *J*_0_ is the extrapolated zero-field critical current density at *T* = 0 K. Value of the exponent, *n*, depends on the structural and electronic anisotropies, nature and distribution of defects, microstructure, level of homogeneity in composition, and details of flux pinning properties^38^. For the sample compositions used in this study, the values of *n* lie within the range 2.00 ± 0.60. The value of the exponent, *n* increases systematically with underdoping. The extracted values of *J*_0_ for different hole contents are shown in Table 1.

**Table 1:** Zero-field and zero-temperature critical current density of Y_1−*x*_Ca_*x*_Ba_2_Cu_3_O_7−*δ*_ thin films.

Compound	Hole content (*p*)	Critical current density, *J*_0_ (10^6^ A/cm^2^)	Normalized critical current density
YBa_2_Cu_3_O_7−*δ*_	0.162	20.62	0.668*
	0.146	14.84	0.481*
	0.102	5.99	0.194*
Y_0.95_Ca_0.05_Ba_2_Cu_3_O_7−*δ*_	0.184	30.88	1.000
	0.170	26.04	0.843
	0.156	22.10	0.716
	0.123	12.10	0.392
Y_0.90_Ca_0.10_Ba_2_Cu_3_O_7−*δ*_	0.198	23.73	0.831
	0.188	28.54	1.000
	0.162	23.50	0.823
	0.160	24.41	0.855
	0.126	13.08	0.458
Y_0.80_Ca_0.20_Ba_2_Cu_3_O_7−*δ*_	0.201	17.08	0.921
	0.186	18.54	1.000
	0.166	17.01	0.917
	0.150	12.98	0.700
	0.144	12.03	0.649
	0.136	10.09	0.544

*Normalized with *J*_0_ = 30.88 × 10^6^ A/cm^2^. See Section 4 for details.

**Figure 3:**
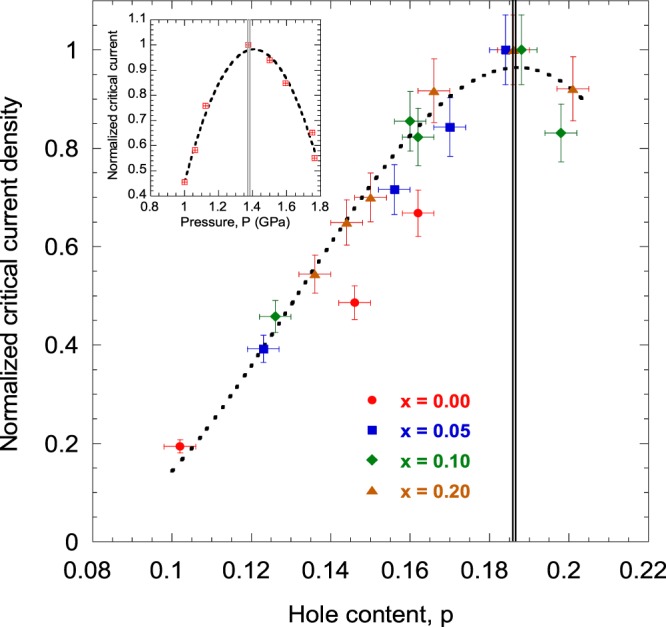
(Main) The normalized zero-temperature and zero-field critical current density of Y_1−*x*_Ca_*x*_Ba_2_Cu_3_O_7−*δ*_ thin films as a function of doped hole content in the CuO_2_ planes. The inset shows the variation of the normalized zero-field critical current of 4.4% Sn-doped CeRhIn_5_ HF superconductor with pressure. The vertical lines mark the maximum critical currents and give the critical hole concentration and the critical pressure, at the putative quantum critical point. The dotted lines are fits to the data drawn as guides to the eyes.

We have plotted the normalized zero-temperature zero-field critical current density for Y_1−*x*_Ca_*x*_Ba_2_Cu_3_O_7−*δ*_ thin films in Fig. 3. *J*_0_(*p*) has been normalized with the maximum value of *J*_0_ for each Ca content (*x*). It is important to note that irrespective of the Ca content and oxygen deficiency in the CuO_1−*δ*_ chain, *J*_0_(*p*) is maximized when *p* ~ 0.185. We have also shown the normalized zero-field critical current for the 4.4% Sn-doped CeRhIn_5_ HF superconductor as a function of pressure (*P*) in the inset. The systematic behavior of doped high-*T*_c_ cuprates and the HF compounds as functions of hole content and pressure are strikingly similar, as far as the critical current is concerned.

**Figure 4:**
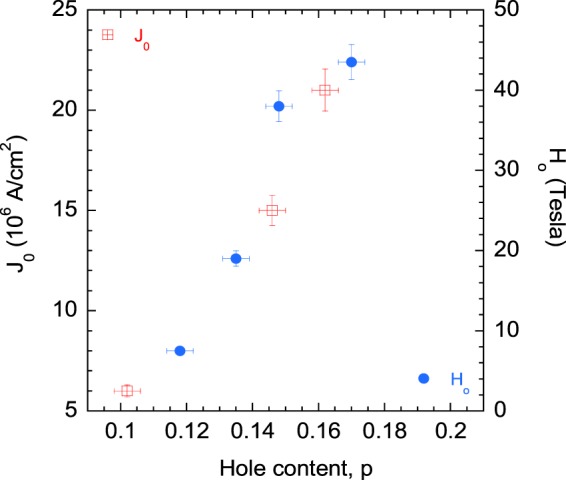
*J*_0_(*p*) and *H*_0_(*p*) of YBa_2_Cu_3_O_7−*δ*_ thin films.

Next, we have shown the *p*-dependent behavior of the characteristic magnetic field, *H*_0_, for YBa_2_Cu_3_O_7−*δ*_ thin films in Fig. 4. *H*_0_(*p*) gives a direct measure of the vortex activation energy and the irreversibility magnetic field^36,38–41^. Resistive broadening of the superconducting transition region as seen in Fig. 2, can be analyzed using the thermally assisted flux flow (TAFF) model^36,41^. The vortex activation energy, or equivalently the vortex pinning energy, *U*(*H*, *T*), can be expressed quite well in a dimensionless form as follows: *U*(*T*, *H*) = (1 − *t*)^*m*^(*H*_0_/*H*)^−*β*^. Here, *t* = *T*/*T*_c_, the reduced temperature, *β* is a constant close to unity, and *m* is an exponent which varies with hole content, anisotropy and nature of the pinning centers within the sample. From the analysis of the *ρ*_*ab*_(*H*, *T*) data for YBa_2_Cu_3_O_7−*δ*_ thin films with different hole concentrations, *H*_0_(*p*) was calculated^36^.

It is worth noting that both *J*_0_(*p*) and *H*_0_(*p*) changes with the doped hole content in the same fashion in YBa_2_Cu_3_O_7−*δ*_. Therefore, it is reasonable to assume that the *p*-dependent zero-field and zero-temperature critical current density in Y_1−*x*_Ca_*x*_Ba_2_Cu_3_O_7−*δ*_ actually reflects the doping dependent vortex activation energy, which in turn is closely linked to the *p*-dependent SC condensation energy and superfluid density of the Cooper pairs^36,42^.

## Discussion and Conclusions

The normalized critical current density as a function of in-plane doped hole content of Y_1−*x*_Ca_*x*_Ba_2_Cu_3_O_7−*δ*_ shows strong resemblance to the normalized critical current of CeRhIn_5_ and 4.4% Sn-doped CeRhIn_5_ HF superconductors as a function of pressure. For the high-*T*_c_ cuprate, critical current density peaks at *p*_c_ ~ 0.185, whereas for the 4.4% Sn-doped CeRhIn_5_ HF compound the critical current peaks for *P*_c_ ~ 1.35 GPa. These particular values of the control parameters are known as the critical hole concentration and critical pressure, respectively. The importance of critical hole concentration in cuprates has been described in several earlier studies^13,21,22,43,44^ in details. The widely investigated pseudogap in the quasiparticle energy spectrum tends to vanish abruptly at this particular hole content^13,21,43,44^, the superfluid density and the superconducting condensation energy is maximized^21,43^, the Fermi surface (FS) goes through a reconstruction^45^, QP peaks appear abruptly in the normal state ARPES spectra^43^, among others.

Quantum criticality describes the collective excitations in strongly correlated systems undergoing a second-order phase transition at zero temperature. How these excitations can lead to formation of Cooper pairs is a matter of intense interest^46,47^. There are strong empirical evidences that spin density wave type quantum criticality can lead to superconductivity^22,24–26^ but a coherent theoretical scheme is yet to be developed. In recent years a number of attempts have been made to formulate quantum critical SC theory to describe the high *T*_c_ and the non-FL behavior of copper oxide superconductors. Wang and Chubukov^20^ have considered spin-mediated superconducting pairing at the antiferromagnetic QCP with an ordering momentum of 2*k*_F_ (*k*_F_ is the Fermi momentum). Kivelson *et al*.^48^ studied the effect of soft critical collective fluctuations at a nematic quantum critical point on superconductivity. It was found that Cooper pairing channel is strengthened by such collective modes. Very recently Abanov *et al*.^49^ considered a quantum-critical metal with interaction mediated by fluctuations of a critical order parameter. This interaction gives rise to two competing tendencies – Cooper pairing and non-Fermi liquid behavior, and seems to reproduce a number of anomalous features seen in the electronic phase diagram of hole doped cuprates. It is important to note that, irrespective of the details and the precise nature of the QCP^19,20,48,49^, all these proposed models predict enhanced superconductivity at the QCP and therefore, provides us with scenarios where the intrinsic critical current density is maximized at the QCP due to its dependence on the SC condensation energy and superfluid density.

For interested readers, some of the basic characteristics of the non-trivial QP excitations in strongly correlated electronic systems arising from the presence of QCPs have been described in greater detail in a related preprint of the current paper in ref.^50^.

As far as the dome shaped *T*_c_(*p*) and *J*_0_(*p*) behaviors for hole doped cuprates are concerned, there are alternative scenarios that can roughly reproduce these features. For example, *t*-*J* model calculations can lead to dome shaped *T*_c_(*p*), and via the estimation of superfluid density, a dome shaped *J*_0_(*p*)^51,52^. One particular drawback of such calculations is that it generally predicts a pseudogap line, the most prominent feature besides *T*_c_(*p*) in hole doped cuprates in the *T*-*p* phase diagram, that merges to the *T*_c_(*p*) line in the overdoped side^51^. Wealth of experimental results^7,12,13,16,21,35,43,44^, on the other hand, indicate that the pseudogap vanishes quite abruptly below the superconducting dome at *p* ~ 0.19 for wide family of hole doped high-T_c_ cuprates^7,12,13,16,21,35,43,44^. This behavior finds strong and natural support within the QCP scenario^53^. In recent times, the pair density wave (PDW) scenario has attracted significant attention^54,55^. Within this particular scheme, the pairing order is periodic in space and fluctuating PDW order exists at high temperatures above *T*_c_^54^. It is interesting to note that advanced theoretical calculations based on single band *t*-*J*-*U* model with charge density wave (CDW) and PDW have shown that a transition between the pure *d*-wave superconducting phase and the coexistent CDW+PDW phase can take place at *p* ~ 0.18 with modulated CDW and PDW orders located in the underdoped regime^55^.

As in the hole doped high-*T*_c_ cuprates and some of the heavy-fermion compounds, superconductivity in iron pnictides emerges in close proximity to the AFM order^22,24,27,56^, and *T*_c_ has dome-shaped dependence on doping or pressure. Electron-doped high-*T*_c_ cuprates are also consistent with the paradigm of an AFM QCP, with AFM order, FS reconstruction, and *T*-linear resistivity all manifested around a QCP at a particular critical doping (*x* = *x*_*c*_) in Nd_2−*x*_Ce_*x*_CuO_4_, Pr_2−*x*_Ce_*x*_CuO_4_, and La_2−*x*_Ce_*x*_CuO_4_^57^. In all these four systems close to the optimal *T*_c_, various normal-state properties show a strong deviation from conventional Fermi liquid behavior. Such remarkable resemblance is highly unlikely to be coincidental. Furthermore, considerable theoretical efforts have been devoted into understanding of the (*ω*/*T*) scaling behavior of the dynamical susceptibility which is thought to be one of the prime features of the existence of an underlying QCP in the electronic phase diagram. Experimental optical conductivity data of optimally hole doped Bi2212 high-*T*_c_ cuprate exhibit such (*ω*/*T*) scaling over an extended region of temperature and energy^58^. This observation supports for quantum critical picture for hole doped cuprates. Therefore, although scenarios alternative to the one based on the QCP exist, the striking resemblance among the electronic phase diagrams of heavy fermions, iron pnictides, electron doped cuprates, and hole doped cuprates makes QCP a very viable framework for comprehensive understanding of the strange normal and superconducting state properties of high-*T*_c_ copper oxide superconductors.

It is worth noticing that in variety of SC systems^22,59^ other than the hole doped cuprates, the quantum critical point coincides with the particular value of control parameter where the SC critical temperature is maximized. In most hole doped cuprates the optimum hole content, *p*_opt_ ~ 0.16, differs from the critical hole concentration, *p*_c_ ~ 0.19. This probably implies that one parameter scaling of quantum critical behavior is probably not adequate^60^ in hole doped cuprates and a separate critical component competing with superconductivity may exist at *p*_c_ ~ 0.19.

It is not surprising to find that *J*_0_ and the characteristic magnetic field *H*_0_ follow the same *p*-dependence for YBa_2_Cu_3_O_7−*δ*_ thin films since *H*_0_ gives a measure of the vortex activation energy^36,38–41^. We predict the similar pressure dependent behavior of the critical current density and vortex pinning energy for pure and doped CeRhIn_5_ HF superconductors. This prediction results from the following arguments. It is reasonable to assume that the flux line is pinned at a site where the SC order parameter is partially or almost completely suppressed. In this situation the pinning energy of the vortex core would reveal itself as the energy barrier to motion of the flux line and therefore, would be equal to the flux activation energy *U*_0_^39^. Here, *U*_0_ denotes the zero temperature activation energy. It is this vortex activation energy that determines the critical current density and the irreversibility magnetic field^36,39^. By a heuristic scaling, Yeshurun and Malozemoff^61^ and Tinkham^62^ have shown that *U*_0_ ~ *H*_c_^2^, where *H*_c_ is the thermodynamical critical magnetic field. The SC condensation energy, *U*_sc_ can also be expressed as *U*_sc_ ~ *H*_c_^2^. Therefore, *U*_0_ ~ *U*_sc_ ~ *H*_c_^2^ ^36,39^. Equivalently, the SC condensation energy can be expressed as *U*_sc_ = *N*(E_F_)Δ_sc_^2^, where *N*(E_F_) is the electronic energy density of states at the Fermi level and Δ_sc_ is the amplitude of the SC spectral gap. The quantity *N*(E_F_)Δ_sc_ measures the Cooper pair number density. The SC coherence gap shows positive correlation with *T*_c_. Therefore, it is logical to assume that in the presence of a QCP where *T*_c_ is maximized as in the case of HF superconductors, the critical current density, thermodynamical critical field and vortex activation energy should also be maximum. At other values of the non-thermal parameter, the variation of these critical current density related parameters should follow the variation in *T*_c_. The arguments presented here are quite general in nature and do not depend significantly on the precise nature of the mechanism leading to Cooper pairing in a particular system.

It is perhaps instructive to notice that even though significant volume of work exists on critical current density of hole doped cuprates, systematic study of critical current density over a wide range of hole content extending from underdoped to overdoped regions of the phase diagram is highly scarce in literature. None of these few systematic studies^30,31,42,63,64^ concerns itself explicitly with possible quantum critical physics in cuprates in relation to heavy fermion superconductors.

It should be noted that we have used the maximum value of *J*_0_ of the 5% Ca substituted compound to normalize the critical current densities of the Ca-free thin film. This is done because these two films show almost similar physical properties including the residual resistivity, slope of the temperature dependent resistivity and SC transition temperature. For example, the maximum *T*_c_ at the optimum hole content (*p* = 0.16) for Y_1−*x*_Ca_*x*_Ba_2_Cu_3_O_7−*δ*_ and YBa_2_Cu_3_O_7−*δ*_ thin films are 92.5 K and 91.0 K, respectively. This possibly introduces a small systematic error in the normalized critical current density of YBa_2_Cu_3_O_7−*δ*_. This error has no significant bearing on the conclusions drawn in this paper.

## Data Availability

The data sets generated and/or analyzed in this study are available from the corresponding author on reasonable request.
